# iPSC‐derived mesenchymal stem cells exert SCF‐dependent recovery of cigarette smoke‐induced apoptosis/proliferation imbalance in airway cells

**DOI:** 10.1111/jcmm.12962

**Published:** 2016-09-19

**Authors:** Xiang Li, Yuelin Zhang, Yingmin Liang, Yuting Cui, Sze C. Yeung, Mary S.M. Ip, Hung‐fat Tse, Qizhou Lian, Judith C.W. Mak

**Affiliations:** ^1^ Department of MedicineThe University of Hong KongHong Kong; ^2^ Department of OphthalmologyThe University of Hong KongHong Kong; ^3^ Research Centre of Heart, Brain, Hormone and Healthy AgingThe University of Hong KongHong Kong; ^4^ Shenzhen Institute of Research and InnovationThe University of Hong KongHong Kong; ^5^ Department of Pharmacology & PharmacyThe University of Hong KongHong Kong

**Keywords:** apoptosis, cigarette smoke, mesenchymal stem cell, proliferation, stem cell factor

## Abstract

Mesenchymal stem cells (MSCs) have emerged as a potential cell‐based therapy for pulmonary emphysema in animal models. Our previous study demonstrated that human induced pluripotent stem cell–derived MSCs (iPSC‐MSCs) were superior over bone marrow–derived MSCs (BM‐MSCs) in attenuating cigarette smoke (CS)‐induced airspace enlargement possibly through mitochondrial transfer. This study further investigated the effects of iPSC‐MSCs on inflammation, apoptosis, and proliferation in a CS‐exposed rat model and examined the effects of the secreted paracrine factor from MSCs as another possible mechanism in an *in vitro* model of bronchial epithelial cells. Rats were exposed to 4% CS for 1 hr daily for 56 days. At days 29 and 43, human iPSC‐MSCs or BM‐MSCs were administered intravenously. We observed significant attenuation of CS‐induced elevation of circulating 8‐isoprostane and cytokine‐induced neutrophil chemoattractant‐1 after iPSC‐MSC treatment. In line, a superior capacity of iPSC‐MSCs was also observed in ameliorating CS‐induced infiltration of macrophages and neutrophils and apoptosis/proliferation imbalance in lung sections over BM‐MSCs. In support, the conditioned medium (CdM) from iPSC‐MSCs ameliorated CS medium‐induced apoptosis/proliferation imbalance of bronchial epithelial cells *in vitro*. Conditioned medium from iPSC‐MSCs contained higher level of stem cell factor (SCF) than that from BM‐MSCs. Deprivation of SCF from iPSC‐MSC‐derived CdM led to a reduction in anti‐apoptotic and pro‐proliferative capacity. Taken together, our data suggest that iPSC‐MSCs may possess anti‐apoptotic/pro‐proliferative capacity in the *in vivo* and *in vitro* models of CS‐induced airway cell injury partly through paracrine secretion of SCF.

## Introduction

Cigarette smoke is the primary cause of chronic obstructive pulmonary disease (COPD) 1, 2, a progressive airway inflammatory disease characterized by persistent airflow obstruction with poor reversibility 3. Emphysema characterized by airspace enlargement, lung parenchyma destruction and elasticity loss is a major cause of the airflow obstruction 3. Chronic obstructive pulmonary disease is predicted to be the fourth leading cause of death by 2030 4, but currently, no pharmacotherapies can reverse the progressive decline of lung function 5. The pathogenesis of COPD involves interactions between oxidative stress, chronic inflammation, excess protease activity and accelerated lung ageing 3, 5, 6. Particularly, emphysema is associated with increased apoptosis of various cell types in the lung, such as endothelial cells, alveolar epithelial cells, fibroblasts and immune cells 7, indicating that the destruction of alveolar wall may be a result of altered apoptosis and proliferation balance.

Mesenchymal stem cells are fibroblast‐like multipotent stem cells existing in various tissues, including bone marrow (BM), amniotic fluid, placenta, adipose tissue and umbilical cord blood 8. Induced pluripotent stem cells (iPSCs) are pluripotent stem cells derived from somatic cells through transcriptional factor‐induced reprogramming 9, 10. Induced pluripotent stem cells can differentiate into MSCs through an induced‐differentiation protocol, providing a new source of MSCs (iPSC‐MSCs) 11. Compared with BM‐MSCs, iPSC‐MSCs have a much higher ability of proliferation without loss of differentiation potential 11 and hold potential to overcome ageing‐associated impairment 12, 13, 14, 15. This may be particularly important for COPD as the prevalence, morbidity and mortality of the disease are highly correlated with age 16, 17, 18. iPSC‐MSCs have been reported to prevent allergic airway inflammation in mice 19. In addition, we have recently demonstrated that iPSC‐MSCs induced a superior effect than BM‐MSCs in attenuating CS‐induced emphysema in rats 20. It is currently unknown whether MSCs could produce beneficial effects through promotion of anti‐apoptotic activity in addition to their anti‐inflammatory activities in our model.

Despite numerous reports of effectiveness of MSCs in diverse disease models, the exact mechanism of their action remains unknown 21. We previously reported that iPSC‐MSCs might work through mitochondrial transfer to lung epithelium 20. Such action requires direct cell contact and is, therefore, restricted to small regions around the MSCs. However, MSCs have been reported to induce paracrine effects *via* release of various immunomodulators 21. The paracrine effect can be effective in a large radius from MSCs as it does not rely on direct cell contact. The role of the paracrine effects is mostly discussed on their immunomodulation, *i.e*. the suppression of inflammation *via* inhibiting immune cells, such as T cells, B cells, dendritic cells and natural killer cells 21. Given the role of apoptosis in the pathogenesis of COPD, airway cell apoptosis may be another site for the paracrine effect to act on. In particular, SCF has been reported to mediate cell survival, migration and proliferation in a cell‐type‐dependent manner through binding to its receptor, tyrosine kinase c‐Kit 22. This study sought to investigate the effects of iPSC‐MSCs on CS‐induced inflammation, apoptosis and proliferation in the rat model using BM‐MSCs for comparison. We hypothesized that iPSC‐MSCs may ameliorate the altered apoptosis/proliferation balance through release of SCF. The paracrine effects will be studied using CdM from iPSC‐MSCs or BM‐MSCs on cigarette smoke medium (CSM)‐treated bronchial epithelial cells *in vitro*.

## Materials and methods

### Preparation of human iPSC‐MSCs, human BM‐MSCs and BEAS‐2B Cells

Human BM‐MSCs (cat. no. PT‐2501; Cambrex Bioscience, Rockland, ME, USA) and immortalized human bronchial epithelial cell BEAS‐2B cells (American Type Culture Collection, Rockville, MD, USA) were obtained commercially. Human iPSC‐MSCs were derived based on a previously published protocol 11. Briefly, IMR90 fibroblast cells (Cat# CCL‐186; American Type Culture Collection, Manassas, VA, USA) were transduced with lentiviral vectors carrying human OCT4, SOX2, NANOG and LIN28 genes (Plasmid 16577‐80; Addgene, Cambridge, MA, USA) followed by incubation with ES culture medium on inactivated mouse embryonic fibroblast feeder for 20 days. Colonies with human embryonic stem cell morphology were identified as iPSCs. For induced‐differentiation, iPSCs were incubated in DMEM (Gibco, Carlsbad, CA, USA) supplemented with 10% serum replacement medium (Gibco), 10 ng/ml basic fibroblast growth factor (bFGF; Gibco), 10 ng/ml platelet‐derived growth factor AB (Peprotech, Rocky Hill, NH, USA), and 10 ng/ml epidermal growth factor (EGF; Peprotech). One week later, differentiating iPSCs were harvested, incubated with CD24‐phycoerythrin and CD105‐FITC (BD PharMingen, San Diego, CA, USA) and sorted by a fluorescence‐activated cell sorting system. The CD24^−^ CD105^+^ cells were sub‐cultured in 96‐well plates to select wells containing a single cell. The clones from a single cell were serially reseeded to obtain a confluent 175‐cm^2^ tissue culture flask. The iPSC‐MSCs were examined for surface marker profile (CD44^+^, CD49a^+^, CD49e^+^, CD73^+^, CD105^+^, CD166^+^, CD34^−^, CD45^−^ and CD133^−^). In addition, the differentiation capacity was tested by efficient adipogenesis, osteogenesis and chondrogenesis. They were then frozen down for future experiments. Both iPSC‐MSCs and BM‐MSCs were cultured in DMEM (Gibco) supplemented with 10% foetal calf serum (Gibco), bFGF (5 ng/ml) and EGF (10 ng/ml). The BEAS‐2B cells were cultured in keratinocyte serum‐free media K‐SFM (Gibco) supplemented with EGF (5 ng/ml) and bovine pituitary extract (50 mg/ml). All cells were maintained in a humidified 37°C incubator with 5% CO_2_.

### MSC treatment of 56‐day CS‐exposed rats

Cigarettes (Camel, filters, 11 mg TAR and 0.8 mg nicotine) (R.J. Reynolds, Winston‐Salem, NC, USA) were obtained commercially. The filters of the cigarettes were removed before the experiment. Male Sprague‐Dawley rats weighted 170–200 g were purchased from the Laboratory Animal Unit, the University of Hong Kong. The rats were treated with 4% (v/v) CS for 1 hr/day for continuous 56 days based on a previously described protocol 23. Briefly, a maximum of eight rats were placed into a ventilated 20‐l‐chamber; 800 ml of 100% CS was injected into the chamber to raise the initial CS concentration to 4%. Then, the chamber was supplied with continuous flow of 4% CS (1 l/min.)—a gas mixture of 100% CS (40 ml/min.) and fresh air (960 ml/min.) from two separate peristaltic pumps (Masterflex; Cole‐Parmer Instruments Co, Niles, IL, USA). The control group is exposed to sham air (SA) by the same protocol. The whole procedure was conducted in a chemical fume hood at the same time of each day. At day 29 and day 43, CS‐exposed rats were intravenously injected with plain PBS, 3 × 10^6^ human BM‐MSCs or 3 × 10^6^ iPSC‐MSCs into tail veins. The SA group was also injected with PBS. No obvious adverse reactions towards the injections were observed based on the daily monitoring of general conditions. The rats were killed 24 hrs after the last CS exposure. Blood was collected *via* cardiac puncture and placed into vacuum tubes without EDTA. Serum was isolated by centrifugation at 1000 × g, 4°C for 10 min. The largest lobe of left lung was firstly inflated with 1 ml formalin and then subjected to fixation, dehydration and paraffin embedding. The procedures were approved by the Committee on the Use of Live Animals in Teaching and Research (CULATR) of the University of Hong Kong (CULATR 3044‐13).

### ELISA and enzyme immune assay

Commercial ELISA kits were used to measure the concentrations of cytokine‐induced neutrophil chemoattractant‐1 (CINC‐1; R&D Systems, Minneapolis, MN, USA) and SCF (R&D Systems) in rat sera and CdM from MSCs. The procedures were based on instructions provided by each manufacturer. Serum 8‐isoprostane levels were measured by 8‐isoprostane express enzyme immune assay kit (Cayman Chemical Company, Ann Arbor, MI, USA) according to manufacturer's protocol. As 8‐isoprostane might be esterified in lipids, all samples were pre‐hydrolysed by incubating with the same volume of 15% (w/v) KOH at 40°C for 60 min. and neutralized with 1 M potassium phosphate to get total 8‐isoprostane levels.

### Generation of CSM

The CSM was prepared according to a previously described method 24. Cigarette smoke of two filter‐removed cigarettes was bubbled into 20 ml of keratinocyte medium without supplements. The medium was filtered through a 0.22‐μm filter and regarded as 100% CSM.

### BM‐MSCs and iPSC‐MSCs CdM treatment of CSM‐treated BEAS‐2B cells

The CdM was prepared as previously described 13. Briefly, BM‐MSCs and iPSC‐MSCs were replaced with DMEM without serum and supplements. After 24 hrs, the medium was collected and concentrated *via* centrifugation by ultrafiltration conical tubes (Amicon Ultra‐15 with membranes selective for 5 kD). The final concentration was adjusted to 20 times that of the collected CdM.

Alternatively, to investigate the effects of SCF, SCF was depleted from iPSC‐MSCs‐CdM as described previously 25. Briefly, 0.5 μg of anti‐SCF (AF‐255‐NA; R&D Systems) or normal human IgG control antibody (1‐001‐A; R&D Systems) were mixed with protein G‐agarose beads in PBS at 4°C for 1 hr with intermittent shaking. After centrifugation, beads were washed three times and used for immune‐depletion of SCF. iPSC‐MSCs‐CdM was incubated with protein G‐agarose beads immobilized with anti‐SCF antibodies or control human antibody for 1 hr at 4°C. Immune complexes absorbed on protein G‐agarose beads were precipitated by centrifugation. Finally, iPSC‐MSCs‐CdM was collected and centrifuged and then used immediately.

The BEAS‐2B cells were cultured on top of coverslips in 24‐well plates. The medium was replaced by keratinocyte medium with no supplements 24 hrs before the treatment. They were then treated with 2% (v/v) CSM. BM‐MSCs‐CdM or iPSC‐MSCs‐CdM containing 3 μg of total protein was added at the same time. After 24 hrs, the supernatant was removed and the cells were fixed for immunohistochemical tests or terminal deoxynucleotidyl transferase‐mediated dUTP nick‐end labelling (TUNEL) assay. To investigate the role of SCF, the cells were treated with SCF‐depleted iPSC‐MSCs‐CdM or recombinant SCF (255‐SC; R&D Systems) with 2% CSM for 24 hrs in comparison with normal iPSC‐MSCs‐CdM.

### Immunohistochemistry and TUNEL assay

Cell apoptosis was determined *via* TUNEL assay using the In Situ Cell Death Detection Kit, POD (Roche Applied Science, Mannheim, Germany). For lung sections, the rehydrated sections were heated in citrate buffer for antigen retrieval, followed by treatment of TUNEL reaction mixture. After 1‐hr incubation in 37°C in dark, the sections were washed and mounted with fluorescent mounting medium with 4′,6‐diamidino‐2‐phenylindole (DAPI) (Prolong Gold antifade reagent with DAPI; Invitrogen, Eugene, OR, USA). For BEAS‐2B cells, cells were fixed with 4% paraformaldehyde in PBS, blocked with 3% H_2_O_2_ in methanol and permeabilized with 0.1% Triton‐100 in 0.1% sodium citrate before treatment of TUNEL reaction mixture. Images of five fields for each slide were captured randomly by a motorized inverted microscope (IX81‐ZDC2; Olympus, Hamburg, Germany) magnification and analysed using AxioVision (Zeiss, Munich, Germany).

For immunohistochemical staining, paraffin‐embedded lung sections were rehydrated, antigen‐retrieved and blocked through the standard procedure. They were then incubated with goat anti‐CD68 (SC‐7084; Santa Cruz, Dallas, Texas, USA), horseradish peroxidase‐conjugated secondary antibody, and 3,3′‐Diaminobenzidine solution in sequence. Alternatively, for immunofluorescent staining, they were incubated with rabbit anti‐Ki67 (ab15580; Abcam, Cambridge, UK), rabbit anti‐neutrophil elastase (NE; ab21595; Abcam) or goat anti‐CC10 (sc‐9772; Santa Cruz), relevant fluorescent secondary antibodies and mounting medium containing DAPI (Invitrogen). As for the BEAS‐2B cells cultured on coverslips, they were fixed with 4% paraformaldehyde in PBS, blocked by 5% bovine serum albumin and permeabilized with 0.1% Triton‐100 in PBS. The cells were then incubated with mouse anti‐cytochrome *c* (SC‐13156; Santa Cruz), rabbit anti‐Ki67 (ab15580) or goat anti‐c‐Kit (SC‐1494; Santa Cruz) followed by relevant fluorescent secondary antibodies. The coverslips were then removed, flipped and put on a drop of mounting medium containing DAPI (Invitrogen) on a glass slide. Images of 10 fields for each slide were captured randomly by a motorized inverted microscope and analysed using AxioVision (Zeiss).

The imaging analysis was performed by counting number of positive cells and the total amount of nuclei in each field. The ratio of positive cells to the total cell number is calculated to avoid the effect of cell density in cell culture or lung sections.

### Western blot

BEAS‐2B cells were scraped and extracted for total protein (CelLytic M mammalian cell lysis/extraction reagent; Sigma‐Aldrich, St. Louis, MI, USA) according to the manufacturer's instructions. The proteins (30 μg) were separated by SDS–polyacrylamide gel and then transferred onto a polyvinylidene fluoride membrane (Bio‐Rad Laboratories, Hercules, CA, USA). After blocking with 5% skim milk in Tris‐buffered saline (pH 7.4) containing 0.1% Tween‐20, the membrane was incubated with diluted goat anti‐c‐Kit (1:500) or mouse anti‐β‐actin (1:5000) at 4°C overnight. The membranes were then incubated with horseradish peroxide‐conjugated anti‐goat antibody (1:2000) or anti‐mouse antibody (1:2000; Dako, Glostrup, Danmark) for 1 hr at room temperature followed by quick incubation with enhanced chemiluminescence. Target proteins were developed as bands on x‐ray films (Fujifilm, Tokyo, Japan).

### Statistical analysis

Numerical data are presented as mean ± S.E.M. Statistical analysis was performed using GraphPad Prism 6.0 (GraphPad Software Inc, San Diego, CA, USA). Differences between groups were evaluated by Student's *t*‐test or one‐way anova followed by Newman–Keuls test when appropriate. Significant difference was defined as when *P* < 0.05.

## Results

### iPSC‐MSCs attenuated CS‐induced macrophage and neutrophil infiltration in the lung of rats

**Figure 1:**
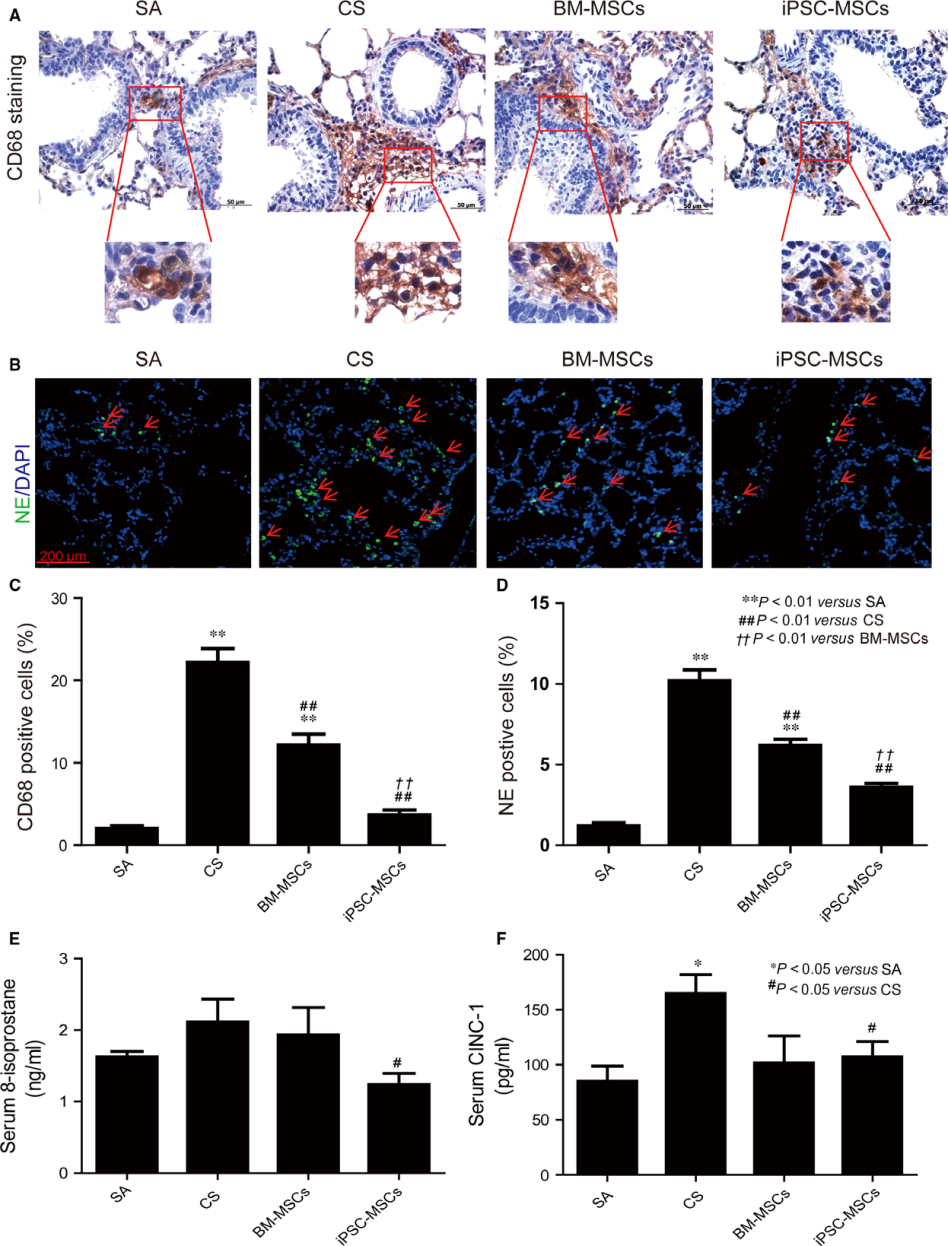
Effects of MSCs on CS‐induced oxidative stress and inflammation in rats. Rats (SA:* n* = 8, CS:* n* = 6, BM‐MSCs: *n* = 7, iPSC‐MSCs: *n* = 7) were exposed to 4% CS 1 hr/day for 56 days. 3 × 10^6^ BM‐MSCs or iPSC‐MSCs were injected at days 29 and 43. (**A**) Tissue macrophages as CD68‐positive cells with brown staining in the lung. Nuclei were stained blue by haematoxylin. (**B**) Tissue neutrophils as neutrophil elastase (NE)‐positive cells with green fluorescence. Nuclei were stained blue with DAPI. (**C**) Ratio of CD68‐positive cells in the lung sections. (**D**) Ratio of NE‐positive cells in the lung sections. (**E**) 8‐isoprostane levels and (**F**) CINC‐1 levels in the serum. *P < 0.05 or **P < 0.01 compared with SA group; ^#^ *P* < 0.05 or ^##^ *P* < 0.01 compared with CS group; ^†^ *P* < 0.05 or ^††^ *P* < 0.01 compared with BM‐MSCs group. Mean ± S.E.M. are shown.

CD68 is a transmembrane glycoprotein highly expressed by monocytes and tissue macrophages 26. In order to illustrate the CS‐induced infiltration of macrophages in lung tissues, immunohistochemistry staining was performed against CD68 in lung sections (Fig. 1A). Cigarette smoke exposure significantly elevated number of macrophages compared with SA group (21.3 ± 1.9 *versus* 2.0 ± 0.41%; *P* < 0.01) (Fig. 1C). Both treatments of iPSC‐MSCs and BM‐MSCs showed significant reduction in the number of macrophages compared with CS group (4.3 ± 0.8% for iPSC‐MSCs and 12.3 ± 1.3% for BM‐MSCs, respectively; *P* < 0.01 in comparison with CS group) with superior effect by iPSC‐MSCs (*P* < 0.01; Fig. 1C).

To examine infiltration of neutrophil in the lung, lung sections were stained for NE (Fig. 1B). Cigarette smoke increased the number of infiltrated neutrophils as NE‐positive cells in the lung (10.2 ± 0.7 *versus* 1.2 ± 0.2%; *P* < 0.01; Fig. 1D). Both BM‐MSCs and iPSC‐MSCs reduced the number of neutrophils (6.2 ± 0.4 and 3.6 ± 0.2% for BM‐MSCs and iPSC‐MSCs, respectively; *P* < 0.01 compared with CS group). iPSC‐MSCs group demonstrated less neutrophils than BM‐MSCs group (*P* < 0.01).

In this study, CS exposure led to a trend of elevation of serum 8‐isoprostane levels but not reaching significance compared with SA group (2.11 ± 0.32 ng/ml *versus* 1.63 ± 0.08 ng/ml) (Fig. 1E). The iPSC‐MSCs treatment significantly reduced the serum levels of 8‐isoprostane compared with the CS group (1.23 ± 0.16 ng/ml for iPSC‐MSCs group, *P* < 0.05). Meanwhile, the BM‐MSCs treatment did not show such attenuation (1.94 ± 0.38 ng/ml for BM‐MSCs group).

Cigarette smoke exposure significantly elevated CINC‐1 levels compared with SA group (164.9 ± 17.13 pg/ml *versus* 85.21 ± 13.56 pg/ml; *P* < 0.05) in serum (Fig. 1F), which was attenuated by treatment of iPSC‐MSCs (107.2 ± 13.84 pg/ml; *P* < 0.05). BM‐MSCs treatment also showed a trend of decrease but not reaching significance (101.8 ± 24.5 pg/ml).

### iPSC‐MSCs reduced apoptosis and promoted proliferation in CS‐exposed rat lung epithelium

**Figure 2:**
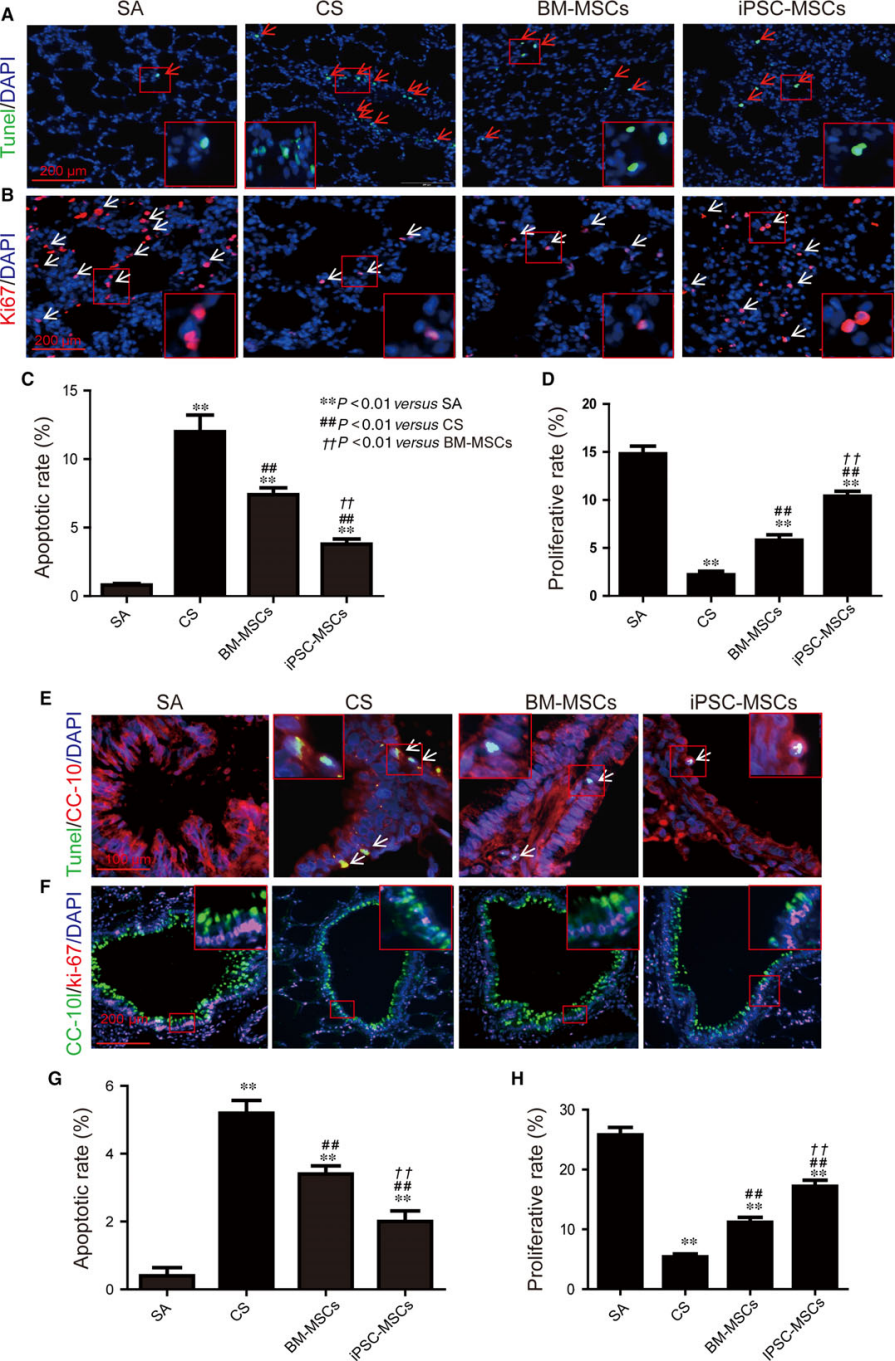
MSCs ameliorated CS‐induced apoptosis/proliferation imbalance in rat lungs. Rats (SA:* n* = 8, CS:* n* = 6, BM‐MSCs: *n* = 7, iPSC‐MSCs: *n* = 7) were exposed to 4% CS 1 hr/day for 56 days. 3 × 10^6^ BM‐MSCs or iPSC‐MSCs were injected at days 29 and 43. (**A**) Apoptotic cells in alveoli of lung sections. Green (TUNEL): apoptotic cells; blue (DAPI): nuclei. (**B**) Proliferative cells in alveoli of lung sections. Red: Ki‐67; blue (DAPI): nuclei. (**C**) Ratio of apoptotic cells in alveolus in lung sections. (**D**) Ratio of proliferative cells in lung sections. (**E**) Apoptotic cells in airway epithelium. Red (CC‐10): airway epithelium; green (TUNEL): apoptotic cells; blue (DAPI): nuclei. (**F**) Illustration of cell proliferation in airway epithelium. Green (CC‐10): airway epithelium; red (Ki‐67): proliferative cells; blue (DAPI): nuclei. (**G**) Ratio of apoptotic cells in airway epithelium. (**H**) Ratio of proliferative cells in airway epithelium. **P < 0.01 compared with SA group; ^##^ *P* < 0.01 compared with CS group; ^††^ *P* < 0.01 compared with BM‐MSCs group. Mean ± S.E.M. are shown.

Cells undergoing apoptosis in alveolar regions of lung sections were labelled green by TUNEL reaction (Fig. 2A). Cigarette smoke treatment significantly increased the number of TUNEL‐positive cells in lung sections (12 ± 2% *versus* 0.8 ± 0.2%; *P* < 0.01; Fig. 2C). The increase was attenuated by both iPSC‐MSCs and BM‐MSCs treatments (3.8 ± 0.8% and 7.4 ± 1%; *P* < 0.01 in comparison with CS group; Fig. 2C). The iPSC‐MSCs group demonstrated significantly fewer number of apoptotic cells than BM‐MSCs group (*P* < 0.01), indicating a better anti‐apoptotic effect of iPSC‐MSCs.

Proliferative cells in alveoli were labelled red by staining against Ki‐67, a marker of proliferation 27 (Fig. 2B). The rate of proliferative cells was significantly reduced by CS exposure compared with SA group (2.2 ± 0.37% *versus* 14.8 ± 0.8%; *P* < 0.01; Fig. 2D). Both BM‐MSCs and iPSC‐MSCs treatments increased rate of proliferative cell compared with CS group (5.8 ± 0.6% and 10.4 ± 0.5% for BM‐MSCs and iPSC‐MSCs, respectively; *P* < 0.01 compared with CS group; Fig. 2D). iPSC‐MSCs group demonstrated higher rate of proliferative cells than BM‐MSCs (*P* < 0.01; Fig. 2D).

Furthermore, we co‐stained TUNEL and Clara cell 10‐kD protein (CC‐10), a marker of epithelium, to determine the apoptosis in epithelium (Fig. 2E). Cigarette smoke exposure increased the number of apoptotic cells in epithelium (5.2 ± 0.7% *versus* 0.4 ± 0.2%; *P* < 0.01), which was reduced by the treatment of BM‐MSCs (3.4 ± 0.5%; *P* < 0.01 in comparison to CS group) or iPSC‐MSCs (2.0 ± 0.6%; *P* < 0.01 in comparison to CS group) (Fig. 2G). The iPSC‐MSCs group also showed significantly lower rate of apoptotic cells in epithelium than BM‐MSCs group (*P* < 0.01; Fig. 2G).

Cigarette smoke exposure also induced a reduction in proliferation in epithelium, which was shown by co‐staining against CC‐10 and Ki‐67 (Fig. 2F). Cigarette smoke exposure reduced the number of proliferative cells in epithelium (5.5 ± 0.5% *versus* 25.8 ± 2.8%; *P* < 0.01), which was ameliorated by the treatment of BM‐MSCs (11.2 ± 0.8%; *P* < 0.01 in comparison to CS group) or iPSC‐MSCs (15.2 ± 2.3%; *P* < 0.01 in comparison to CS group; Fig. 2H). The iPSC‐MSCs group demonstrated higher proliferative activity in epithelium than the BM‐MSC group (*P* < 0.01; Fig. 2H).

### iPSC‐MSCs‐CdM reduced apoptosis and promoted proliferation in CSM‐treated BEAS‐2B cells

**Figure 3:**
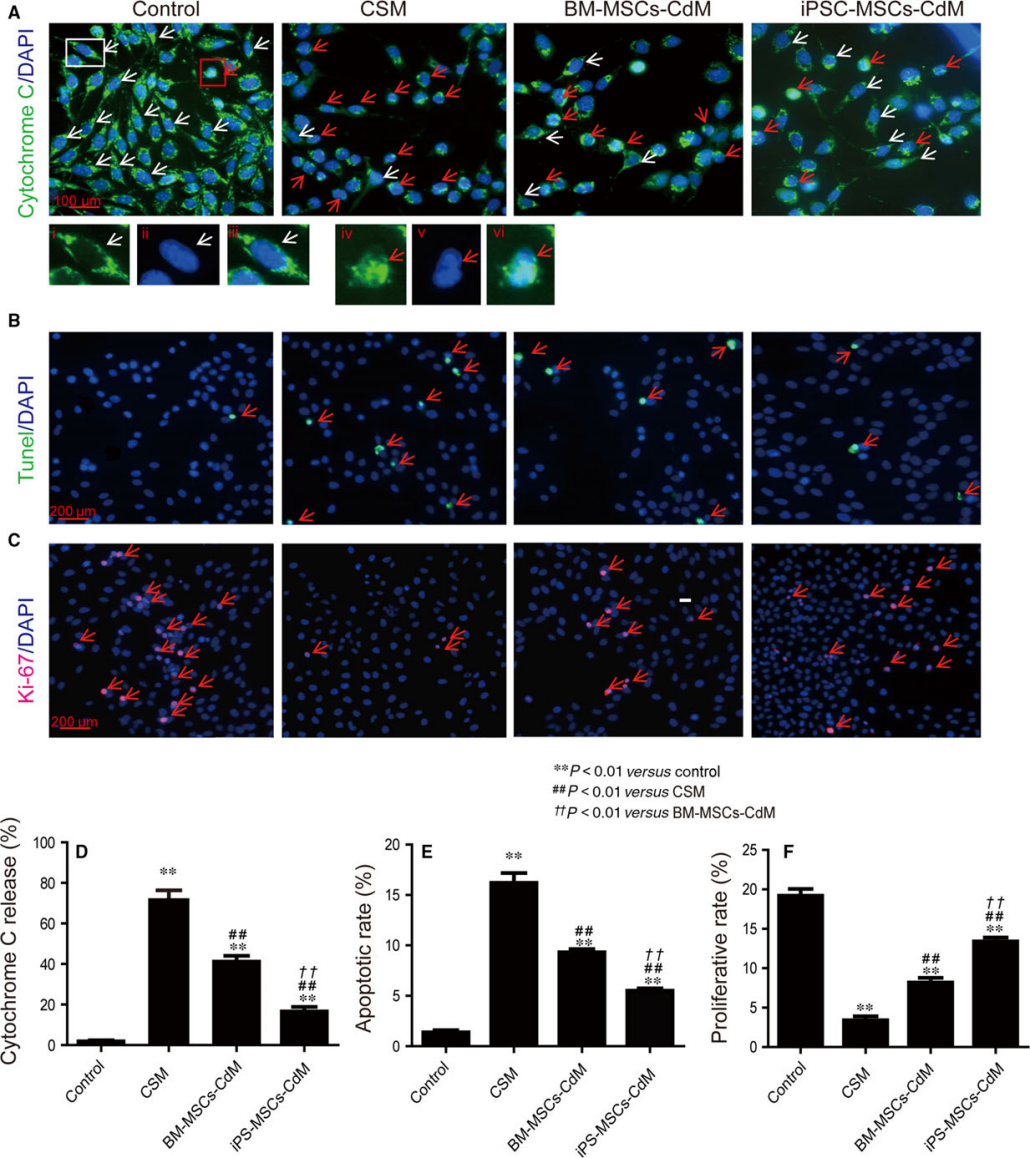
CdM from BM‐MSCs and iPSC‐MSCs ameliorated CSM‐induced apoptosis/proliferation imbalance in BEAS‐2B cells. BEAS‐2B cells were treated with 2% CSM and BM‐MSCs‐CdM or iPSC‐MSCs‐CdM for 24 hrs. (**A**) Cytochrome *c* translocation in BEAS‐2B cells. Green: cytochrome *c*; blue: DAPI. Cells with cytochrome *c* translocation to nuclei are marked by red arrows and cells without cytochrome *c* translocation to nuclei are marked by white arrows. (i) Cytochrome *c* channel, (ii) DAPI channel and (iii) merged image of a healthy cell. It is clear that the nucleus is negative for the cytochrome *c* staining. (iv) Cytochrome *c* channel, (v) DAPI channel and (vi) merged image of a cell with nuclear translocation of cytochrome *c*. It is clear that the nucleus region is now also positive for cytochrome *c*. (**B**) Apoptotic BEAS‐2B cells as stained by TUNEL. Green: TUNEL; blue: DAPI. (**C**) Proliferative BEAS‐2B cells as marked by Ki‐67. Red: Ki‐67; blue: DAPI. (**D**) Percentage of BEAS‐2B cells with cytochrome *c* translocation (*n* = 3). (**E**) Percentage of apoptotic BEAS‐2B cells (*n* = 3). (**F**) Percentage of proliferative BEAS‐2B cells. **P < 0.01 compared with control group; ^##^ *P* < 0.01 compared with CSM group; ^††^ *P* < 0.01 compared with BM‐MSCs‐CdM group. Mean ± S.E.M. are shown.

BEAS‐2B cells were treated with 2% CSM and CdM for 24 hr, and cell apoptosis was determined by TUNEL assay (Fig. 3B). The 2% CSM treatment increased the percentage of apoptotic cells (16.2 ± 2% *versus* 1.4 ± 0.4%; *P* < 0.01), which was attenuated by both BM‐MSCs‐CdM (9.3 ± 0.7%; *P* < 0.01 in comparison to CSM group) and iPSC‐MSCs‐CdM (5.5 ± 0.5%; *P* < 0.01 in comparison to CSM group; Fig. 3E). The iPSC‐MSCs‐CdM group exhibited fewer apoptotic cells than BM‐MSCs‐CdM group (*P* < 0.01), indicating a better anti‐apoptotic effect of iPSC‐MSCs‐CdM. The release of cytochrome *c* from mitochondria and its translocation to nucleus marks a major event leading to the onset of apoptosis 28, 29, 30. In this study, the percentage of cells with cytochrome *c* translocation was significantly increased by the 2% CSM treatment (73.4 ± 6.8% *versus* 1.88 ± 1%; *P* < 0.01; Fig. 3A and D), which was attenuated by either BM‐MSCs‐CdM (41.2 ± 6.3%; *P* < 0.01 in comparison to CSM group) or iPSC‐MSCs‐CdM (16.6 ± 5%; *P* < 0.01 in comparison to CSM group; Fig. 3D). iPSC‐MSCs‐CdM demonstrated a greater effect compared with BM‐MSCs‐CdM (*P* < 0.01; Fig. 3D).

In addition to the effect on apoptosis, 2% CSM treatment also significantly reduced the proliferation of the cells, indicated by significantly reduced Ki‐67‐positive cell number in the population (3.4 ± 0.5% *versus* 19.2 ± 0.8%; *P* < 0.01; Fig. 3C and F). Both BM‐MSCs‐CdM (8.2 ± 0.6%; *P* < 0.01 in comparison to CSM group) and iPSC‐MSCs‐CdM (13.4 ± 0.5%; *P* < 0.01 in comparison to CSM group) were able to reverse such reduction (Fig. 3F). Again, iPSC‐MSCs‐CdM showed a superior effect than BM‐MSCs‐CdM (*P* < 0.1; Fig. 3F).

### Amelioration of CSM‐induced apoptosis/proliferation imbalance was SCF‐dependent in BEAS‐2B cells

**Figure 4:**
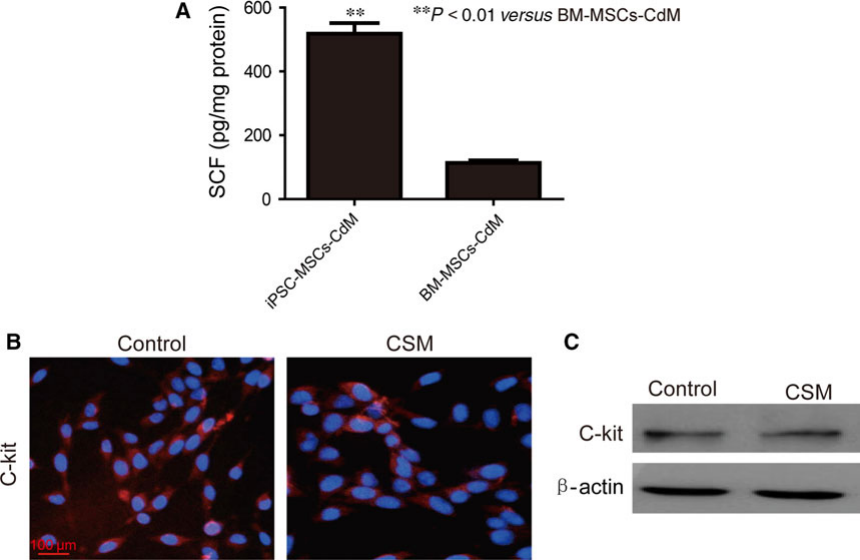
Secretion of SCF by BM‐MSCs and iPSC‐MSCs and expression of c‐Kit by BEAS‐2B cells. (**A**) SCF levels in CdM from BM‐MSCs or iPSC‐MSCs as measured by ELISA (*n* = 3). (**B**) Illustration of c‐Kit in BEAS‐2B cells as identified by immunofluorescent staining with or without 2% CSM treatment for 24 hrs. (**C**) c‐Kit levels in BEAS‐2B cells by Western blotting. Red: c‐Kit; blue: DAPI. **P < 0.01 compared with BM‐MSCs‐CdM group. Mean ± S.E.M. are shown.

Given the superior anti‐apoptotic and pro‐proliferative effect of iPSC‐MSCs‐CdM than BM‐MSCs‐CdM, we further elucidated the components in the CdM that induced such an effect. In particular, we were interested in SCF which has been demonstrated to attenuate vascular smooth muscle apoptosis 31. In this case, the concentration of SCF in CdM was determined, showing that iPSC‐MSCs released much higher level of SCF than BM‐MSCs (518 ± 57 *versus* 113 ± 13 pg/mg protein; *P* < 0.01; Fig. 4A). The expression of SCF receptor, c‐Kit, was also observed *via* immunofluorescent staining (Fig. 4B) and Western blotting (Fig. 4C) in BEAS‐2B cells. The data illustrated that expression of c‐Kit did not alter after CSM treatment (Fig. 4C).

**Figure 5:**
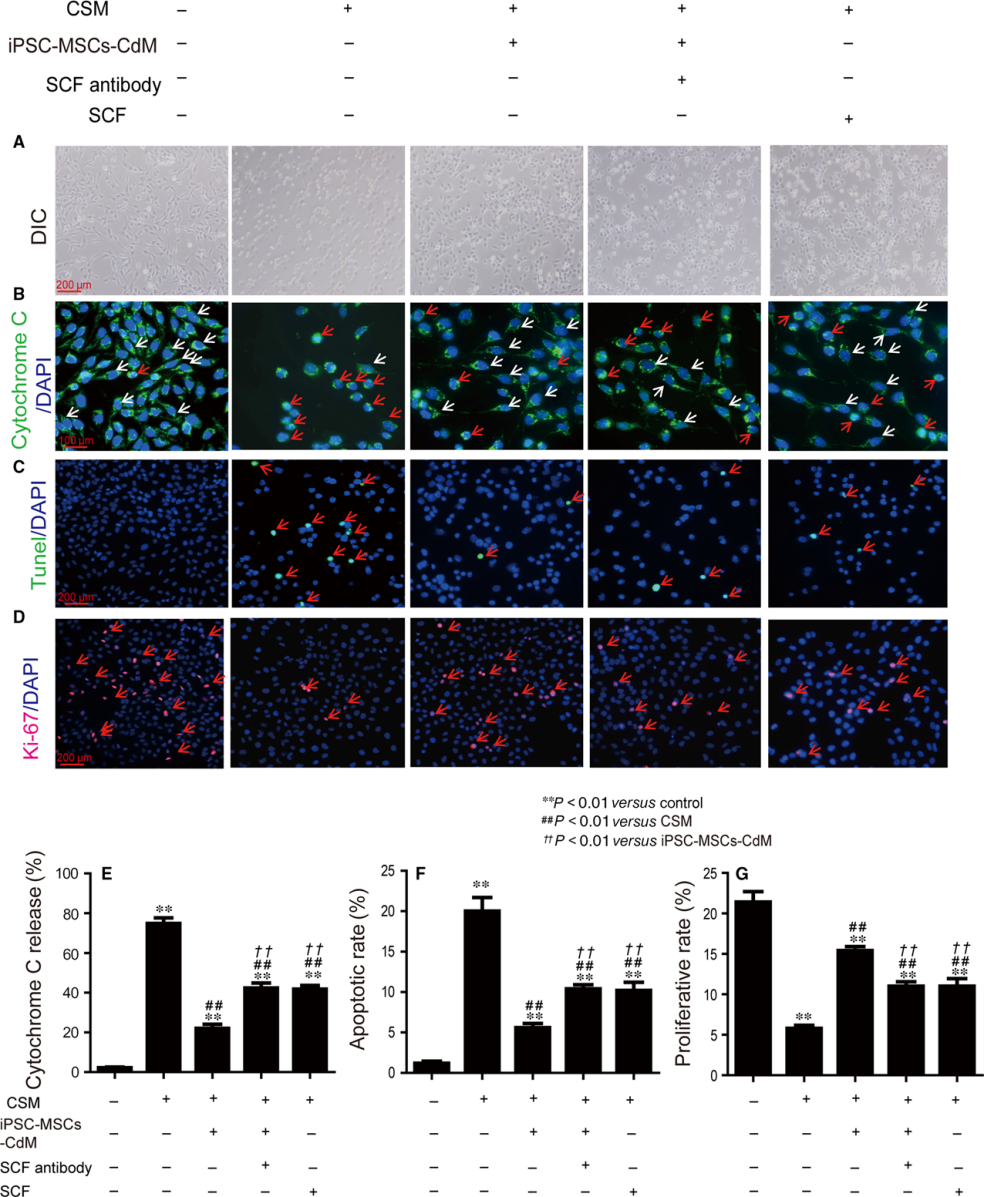
The anti‐apoptotic and pro‐proliferative activity of iPSC‐MSCs‐CdM was partly through SCF. Immobilized anti‐SCF antibody was used to scavenge SCF from iPSC‐MSCs‐CdM. BEAS‐2B cells were treated with 2% CSM and iPSC‐MSCs‐CdM, SCF‐deprived iPSC‐MSCs‐CdM or human recombinant SCF for 24 hrs before fixation and TNUEL staining or immunofluorescent staining against cytochrome *c* and Ki‐67 (*n* = 3). (**A**) The morphological change of the BEAS‐2B cells. DIC: differential interference contrast. (**B**) Cytochrome *c* translocation in BEAS‐2B cells. Green: cytochrome *c*; blue: DAPI. Cells with cytochrome *c* translocation to nuclei are marked by red arrows, and cells without cytochrome *c* translocation to nuclei are marked by white arrows. (**C**) Apoptotic BEAS‐2B cells as stained by TUNEL. Green: TUNEL; blue: DAPI. (**D**) Proliferative BEAS‐2B cells as marked by Ki‐67. Red: Ki‐67; blue: DAPI. (**E**) The percentage of BEAS‐2B cells with cytochrome *c* translocation to nuclei (*n* = 3). (**F**) The percentage of apoptotic BEAS‐2B cells (*n* = 3). (**G**) The percentage of proliferative BEAS‐2B cells (*n* = 3). **P < 0.01 compared with control group; ^##^ *P* < 0.01 compared with CSM group; ^††^ *P* < 0.01 compared with normal iPSC‐MSCs‐CdM group. Mean ± S.E.M. are shown.

To elucidate the role of SCF, SCF in iPSC‐MSCs‐CdM was scavenged by SCF antibody. BEAS‐2B cells were treated with 2% CSM together with iPSC‐MSCs‐CdM, SCF‐deprived iPSC‐MSCs‐CdM or human recombinant SCF for 24 hrs. All of the three treatments attenuated CSM‐induced morphological change (Fig. 5A), cytochrome *c* translocation (22.0 ± 2.1%, 42.4 ± 2.5% and 41.8 ± 1.9% for iPSC‐MSCs‐CdM, SCF‐deprived group, and recombinant SCF group, respectively, *P* < 0.01 for each group compared to CSM group 74.8 ± 2.9%; Fig. 5B and E), apoptosis (5.6 ± 0.5%, 10.4 ± 0.5% and 10.2 ± 1.0%, respectively, *P* < 0.01 compared to CSM group 20 ± 1.7%; Fig. 5C and F) and reduction in proliferation (15.4 ± 0.5%, 11.0 ± 0.5% and 11.0 ± 0.9%, respectively, *P* < 0.01 compared to CSM group 5.8 ± 0.4%; Fig 5D and G). Compared with the iPSC‐MSCs‐CdM treatment, SCF‐deprived iPSC‐MSCs‐CdM led to significantly weakened activity, as indicated by more cytochrome *c* translocation (*P* < 0.01), more apoptosis (*P* < 0.01) and less proliferation (*P* < 0.01). This observation, in combination with the finding that human recombinant SCF was able to induce considerable anti‐apoptotic and pro‐proliferative effects by itself, suggested that SCF plays a role in the activity of iPSC‐MSCs‐CdM. Nevertheless, the human recombinant SCF was not as effective as the iPSC‐MSCs‐CdM regarding cytochrome *c* translocation (*P* < 0.01), apoptosis (*P* < 0.01) and proliferation (*P* < 0.01). Given that the SCF‐deprived iPSC‐MSCs‐CdM still retained some capacity to restore all three parameters, these data indicated that in addition to SCF, there should be other components promoting similar effects. The action of iPSC‐MSCs‐CdM to alleviate CSM‐induced apoptosis/proliferation imbalance was partly dependent on SCF in BEAS‐2B cells.

## Discussion

This study demonstrated that iPSC‐MSCs could ameliorate CS‐induced macrophage/neutrophil infiltration and apoptosis/proliferation imbalance in rat lungs, in line with the attenuation of CSM‐induced apoptosis and the reversal of reduction in proliferation using iPSC‐MSCs‐CdM in bronchial epithelial cells *in vitro*. Deprivation of SCF from iPSC‐MSCs‐CdM led to reduced efficacy, indicating that the action was partly mediated by SCF.

Currently, no medication is able to attenuate the progressive decline of lung function in COPD 32. As COPD is predicted to be the fourth leading cause of death by 2030 4 associated with enormous economic and social burden, seeking new treatments for COPD is an urgent and important task. MSCs from BM, adipose or cord blood have been demonstrated to attenuate emphysema induced by CS, elastase or VEGF deficiency 21, 33, 34, 35, 36, 37. This led to a double‐blind, placebo‐controlled phase II clinical trial of systemic administrations of BM‐MSCs to moderate or severe COPD patients 38. However, BM‐MSCs did not improve lung function or quality of life indicators of the patients compared with the placebo group in the trial. The results point out the possibility that BM‐MSCs might not be effective in the clinical setting, despite the efficacy reported in various animal models. Such findings call for searching for improved MSCs therapies. After all, the trial demonstrated great safety of BM‐MSCs in patients with moderate or severe COPD in the 2‐year follow‐up, leaving the door open for future trials.

The *in vitro* differentiation of iPSCs into MSCs provides a new source of MSCs, which brings potential opportunities to overcome several limitations of adult MSCs as seen in BM‐MSCs. First, iPSC‐MSCs have a greater expansion capacity, maintaining differentiation potential and normal karyotypes for 120 doublings, while BM‐MSCs get senescent after 20 doublings 11. Second, BM‐MSCs may present reduced survival and differentiation ability when isolated from aged subject or patients with ageing‐related disorders 12, 13, 14, 15. iPSC‐MSCs may overcome the ageing‐associated impairment of BM‐MSCs, which may be important in COPD wherein the prevalence, morbidity and mortality are related to age 16, 17, 18. To generate iPSC‐MSCs, somatic cells were first reprogrammed to an embryonic stem cell‐like state, which may help to rejuvenate the cells and overpass the problem of senescence. Indeed, the telomerase activity of iPSC‐MSCs is 10 times higher compared with BM‐MSCs 11. Third, individual adult MSC types like BM‐MSCs may still present a heterogeneous group of cells of different subtypes 39. This intra‐population heterogeneity is a limitation of MSCs‐based therapy as current understanding of functional attributes of MSCs at the population level is still limited 39. The iPSC‐MSCs may represent a more homogenous population as they grew from a single iPSC‐MSC. Our study represented a comparison between a preparation of iPSC‐MSCs and a preparation of BM‐MSCs. It is hard to tell if the superior effect of iPSC‐MSCs was due to the higher ‘purity’ of active MSCs. But such comparison mimics a comparison of potential therapeutic applications, because the same issue exists in a clinical context. We have previously demonstrated that iPSC‐MSCs possessed a superior capacity than BM‐MSCs in attenuating CS‐induced emphysema in rats 20.

The time‐points chosen in the animal model of this study were based on our previous study 20. As 28‐day CS exposure can induce moderate lung damage already, the rats were exposed to CS alone but received no MSCs treatment, mimicking clinical situation as an intervention. Based on our pilot study, CS cessation would recover a few parameters used to define the lung damage in the model. Therefore, the CS exposure was kept for another 28 days during the MSCs treatment to maintain the oxidative stress and inflammation. In the clinical trial of MSCs in COPD, the patients received MSCs infusion every month for 4 months 38, while in another clinical trial of MSCs in pulmonary fibrosis, MSCs were given every week for 1 month 40, in line with the frequency of injection in this study.

Mesenchymal stem cells express very low levels of major histocompatibility complex class I and class II molecules, which makes them highly immunologically tolerant 33, 41, 42, 43. Exogenous administration of allogeneic MSCs usually leads to low levels of immune rejections and does not require immunosuppression. In this study, human MSCs were injected into rats and no immunological rejection was observed 8. The efficacy of human iPSC‐MSCs in rodent models of limb ischaemia, allergic inflammation and emphysema was observed. It has also been reported by other groups that both mouse and human adult adipose MSCs were able to attenuate emphysema in mouse models 37. Despite the use of humanized mouse model, the study proved that human MSCs worked very well on mouse tissues. Based on the immune‐tolerant property of MSCs, it may not be necessary to avoid immunological rejection by using the humanized model.

Chronic inflammation is profiled in COPD especially in the small airways 44, and the severity of the disease is correlated to the degree of inflammation 45. In this study, we demonstrated better efficacy of iPSC‐MSCs to reduce CS‐induced infiltration of macrophages and neutrophils in comparison to BM‐MSCs. Interleukin (IL)‐8, a potent chemoattractant for neutrophil recruitment 46, is a biomarker of neutrophilic inflammation in COPD, with increased levels in sputum and plasma in COPD patients in correlation to the severity 47. Cytokine‐induced neutrophil chemoattractant‐1 is the rat homologue to human IL‐8, acting as a major neutrophil chemoattractant and activator in rats 48, 49. In this study, iPSC‐MSCs were able to attenuate the CS‐induced elevation of serum CINC‐1 levels. As inflammation is associated with oxidative stress in COPD, we further measured the serum levels of 8‐isoprostane, a by‐product of lipid peroxidation and a biomarker of oxidative stress 50. Elevated 8‐isoprostane levels in breath and urine have been found among patients with COPD 51, 52. In this study, iPSC‐MSCs, but not BM‐MSCs, reduced serum 8‐isoprostane in comparison to CS group, indicating that the effect of iPSC‐MSCs on macrophage/neutrophil infiltration might be associated with effect on oxidative stress.

There are now mounting data suggesting that disturbance of the balance between apoptosis and proliferation plays an important role in the gradual destruction of alveolar structure in COPD 7. Cigarette smoke is reported to affect apoptosis of various cell types in the lung, such as endothelial cells, alveolar epithelial cells, fibroblasts and immune cells 7. Excess apoptosis in alveolar cells of patients with no compensation from increased proliferation results in the gradual destruction of alveolar walls and finally leads to emphysema 7. In some animal models, emphysema can be induced by apoptosis of alveolar wall and endothelial cells, even without the infiltration of inflammatory cells 7. In our previous study, iPSC‐MSCs attenuated CS‐induced emphysema in rats 20. Here, we further demonstrated that CS exposure led to increased apoptosis and reduced proliferation in lung epithelium, which was alleviated by treatment with iPSC‐MSCs or BM‐MSCs. iPSC‐MSCs demonstrated superior anti‐apoptotic and pro‐proliferative effects in comparison to BM‐MSCs.

Currently, there is only limited understanding about the mechanism of MSCs' action 21. We previously reported mitochondrial transfer to epithelium as one mechanism 20. The limitation of this mechanism is that mitochondrial transfer relies on direct contact between iPSC‐MSCs and airway cells. Although we observed the retention of MSCs in lung even 2 weeks after injection 20, it is unlikely that they can reach every cell to take effect. The paracrine effect of MSCs has been documented in various studies, many active factors being identified, such as VEGF, transforming growth factor‐β_1_, hepatic growth factor, basic FGF‐ and platelet‐derived growth factor 21. While most studies of paracrine effects were focused on immunoregulation 21, one previous report demonstrated that BM‐MSCs reduced CSM‐induced apoptosis in a human umbilical vein endothelial cell line partly through release of VEGF 34. In our current study, CdM from MSCs reduced the CSM‐induced apoptosis of BEAS‐2B cells, which might result from the attenuation of CSM‐induced cytochrome *c* release from mitochondria and its translocation, an event that is reported to initiate cellular apoptosis 28, 29, 30. This implies that MSCs may be able to attenuate CS‐induced cytochrome *c* translocation in the lung epithelium, leading to attenuation of CS‐induced apoptosis seen in our *in vivo* model. This is in support of our previous report that MSCs attenuate CS‐induced airspace enlargement in rat lung 20. While apoptosis was induced by CS, the proliferation activity was reduced in BEAS‐2B cells, indicating an imbalance of the equilibrium between apoptosis and proliferation. The CdM from MSCs reversed the CSM‐induced reduction in proliferation, leading to the restoration of the apoptosis/proliferation imbalance. iPSC‐MSCs‐CdM demonstrated higher ability than BM‐MSCs‐CdM in all the observed effects, suggesting a higher anti‐apoptotic and pro‐proliferative capacity of iPSC‐MSCs through paracrine action.

Stem cell factor is a dimeric protein that can mediate cell survival, migration and proliferation through binding to its receptor, tyrosine kinase c‐Kit 22. The SCF/c‐Kit signalling is essential to maintain normal alveolar structure in mice. Deficiency in c‐Kit signalling is reported to result in spontaneous airspace enlargement, increased *ex vivo* lung compliance and enlarged residual volume in mice 53. In this study, BM‐MSCs and iPSC‐MSCs secreted SCF with the presence of c‐Kit in BEAS‐2B cells whose level of c‐Kit was not changed by CSM treatment. We found that SCF by itself was able to attenuate CSM‐induced apoptosis, cytochrome *c* translocation and reduction in proliferation in BEAS‐2B cells, while deprivation of SCF significantly reduced capacity of iPSC‐MSCs‐CdM to achieve such effects. Such data suggest that SCF plays an important role in the action of CdM. However, the effects of SCF alone were much weaker than iPSC‐MSCs‐CdM, and the SCF‐deprived iPSC‐MSCs‐CdM was still capable to reduce the apoptosis and restore the proliferation, indicating that the action of iPSC‐MSC‐CdM was only partly through SCF. In addition, iPSC‐MSCs secreted significantly higher level of SCF compared with BM‐MSCs, which may explain the superior anti‐apoptotic and pro‐proliferative effects of iPSC‐MSCs‐CdM.

Nevertheless, there are some limitations in this study. First, the *in vivo* study may not be able to fully mimic the clinic scenario because of the cross‐species nature. Developing protocols for derivation of rat iPSC‐MSCs and testing their effects in the rat model may be a future step. Second, in the *in vivo* study*,* as the measurements for inflammation and oxidative stress are not comprehensive before looking into apoptosis/proliferation imbalance, the status of oxidative stress and inflammation can be further investigated. Third, CdM was directly collected from normal culture of iPSC‐MSCs or BM‐MSCs which did not have interactions with the BEAS‐2B cells. However, in a paracrine crosstalk, BEAS‐2B cells may release cytokines that can act on MSCs to affect secretion of molecules from MSCs. In the *in vivo* model, when MSCs circulated around the body, they will also interact with the microenvironment around them. Such effects need to be further investigated. Lastly, this study showed the contribution of SCF to the anti‐apoptotic and pro‐proliferative effects, but the deprivation of SCF did not completely eliminate the anti‐apoptotic and pro‐proliferative activity of the CdM, suggesting the involvement of other active molecules that need to be determined in the future.

In conclusion, our data indicated that SCF partly mediated the activity of iPSC‐MSCs to ameliorate CS‐induced apoptosis/proliferation imbalance in airway cells through paracrine effects. The higher level of SCF in iPSC‐MSCs‐CdM over BM‐MSCs‐CdM may explain the better anti‐apoptotic and pro‐proliferative capacity in the *in vivo* and *in vitro* models of CS‐induced airway cell injury. Our findings further support iPSC‐MSCs as a promising candidate in cell‐based therapy for the intervention of smoking‐related COPD.

## Conflicts of interest

All authors declare no financial conflicts of interest.

## Author contribution

Conception and design: X.L., Y.Z., Q.L. and J.C.W.M.; data acquisition: X.L., Y.Z., Y.L, Y.C. and S.C.Y.; analysis and interpretation: X.L. and Y.Z.; manuscript drafting: X.L.; manuscript revising for submission: X.L., Y.Z., Q.L., H.T., M.S.M.I. and J.C.W.M.
